# Histomorphological Changes in Fish Gut in Response to Prebiotics and Probiotics Treatment to Improve Their Health Status: A Review

**DOI:** 10.3390/ani13182860

**Published:** 2023-09-08

**Authors:** Giuseppe De Marco, Tiziana Cappello, Maria Maisano

**Affiliations:** Department of Chemical, Biological, Pharmaceutical and Environmental Sciences, University of Messina, 98166 Messina, Italy; gdemarco@unime.it (G.D.M.); mmaisano@unime.it (M.M.)

**Keywords:** fish gastrointestinal tract (GIT), feed additives, microbial communities, fish welfare assessment, histological assays, aquaculture

## Abstract

**Simple Summary:**

Activities such as the digestion and absorption of feeds occur into the gastrointestinal tract (GIT), which also serves to excrete waste products of digestion. These processes occur thanks to the different species of microorganisms inhabiting the GIT, the microbiota, which contribute to the health status of fish by providing metabolic benefits and counteracting pathogen infection. The microbiota is affected by environmental conditions and by the dietary habits of fish species, and it may be modulated by the administration of feed additives based on prebiotics and probiotics. These represent a very useful tool to improve the health status of fish since they are able to enhance gut efficiency, nutrient uptake, defense against pathogens, and growth performance, as may also be assessed by histological endpoints. Overall, a better understanding of the appropriate administration of feed supplements for individual fish species is a promising strategy for improving of the quality and sustainability of the aquaculture sector.

**Abstract:**

The gastrointestinal tract (GIT) promotes the digestion and absorption of feeds, in addition to the excretion of waste products of digestion. In fish, the GIT is divided into four regions, the headgut, foregut, midgut, and hindgut, to which glands and lymphoid tissues are associated to release digestive enzymes and molecules involved in the immune response and control of host-pathogens. The GIT is inhabited by different species of resident microorganisms, the microbiota, which have co-evolved with the host in a symbiotic relationship and are responsible for metabolic benefits and counteracting pathogen infection. There is a strict connection between a fish’s gut microbiota and its health status. This review focuses on the modulation of fish microbiota by feed additives based on prebiotics and probiotics as a feasible strategy to improve fish health status and gut efficiency, mitigate emerging diseases, and maximize rearing and growth performance. Furthermore, the use of histological assays as a valid tool for fish welfare assessment is also discussed, and insights on nutrient absorptive capacity and responsiveness to pathogens in fish by gut morphological endpoints are provided. Overall, the literature reviewed emphasizes the complex interactions between microorganisms and host fish, shedding light on the beneficial use of prebiotics and probiotics in the aquaculture sector, with the potential to provide directions for future research.

## 1. Introduction

In teleosts, the gastrointestinal tract (GIT) consists of a continuous hollow tube that opens to the outside at both ends, through the mouth at one end and through the anus at the other. Along this structure, it is possible to recognize areas that differ in histology and function since several activities occur, including digestion, absorption, and excretion of waste materials. As in mammals, a number of endogenous enzymes and key molecules produced by associated glands, such as the liver, pancreas, and cells of the intestinal wall, are released into the GIT to contribute to all of these actions [1]. The role of these enzymes is coupled with a variety of fermentation processes carried out by microorganisms (bacteria, fungi, and yeasts) colonizing the GIT that constitute the so-called “microbiota”, which has co-evolved with the host in a symbiotic relationship and is responsible for providing metabolic benefits and counteracting pathogen infection.

In fish, the number of microorganisms inhabiting the GIT has been estimated in the range of 10^7^–10^8^ per gram [2]. However, the GIT microbiota is highly variable and its normal variation in abundance and diversity of microorganisms is affected by several factors [3] including age, diet, host genetics, and the environment (freshwater, seawater). Feeding habits are surely one of the most relevant factors affecting the composition of the GIT microbiota among fish species. Indeed, it is well known how different dietary habits (herbivores, carnivores, omnivores, detritivores) come together with variations in both fish GIT morphology and microbial populations [3,4,5]. The strict correlation between the structures of the digestive apparatus and the feeding habit of fishes [6], as revealed in some morphometric parameters such as the intestinal length and area, highly influences the microbial populations detected along the alimentary canal [7]. On the other hand, microorganisms may also affect the digestive morphological structures and functions, both negatively [8], and positively, as in the case of probiotics [9].

In accordance with the currently adopted definition by the Food and Agricultural Organization of the United Nations (FAO) and the World Health Organization (WHO), probiotics are live microorganisms that, when administered in adequate amounts, confer health benefits to the host [10]. In fact, after administration, probiotics are able to colonize and multiply in the gut of the host and enact numerous beneficial effects by modulating various biological systems in the host [11,12], including immune status, growth performance, disease resistance, and feed conversion, with an overall improvement of the fish health status [13,14]. Probiotics, together with other additives such as prebiotics, which are referred to as the food or energy provider for good microorganisms [15], aim to optimize the host–microbiota ratio, which plays a key role in ensuring the proper functioning of the fish GIT [3,16]. Therefore, for all the above-mentioned reasons, the use of these feed additives has gained a key strategic role in the aquaculture sector [14,16].

Overall, this review focuses on the current knowledge regarding the use of a variety of probiotics and prebiotics in different fish species for the improvement of their health and gut efficiency. Furthermore, particular attention was paid to the mutual influences between the microbial populations and morphological features of the fish GIT areas, especially of the gut tract, which is usually investigated by histological assays to evaluate the performance of various feed additives in aquaculture.

## 2. The Gastrointestinal Tract (GIT) in Fish

As mentioned above, along the “tube”-termed GIT, it is possible to recognize different areas distinguishable from a functional and histological point of view. The main areas of the fish GIT can be summarized as follows:

- Headgut: the area where it is possible to distinguish between a buccal and a pharyngeal cavity;
- Foregut: the area that begins at the posterior edge of the gills and includes the oesophagus, the stomach, and the pylorus;
- Midgut: the anterior intestine, which includes a variable number of pyloric caeca or appendages which are useful for increasing the surface area and optimizing the absorption of nutrients;
- Hindgut: the area including the distal intestine and the anus.

The headgut, mainly characterized by the mouth cavity, plays the key role of ensuring feed acquisition; therefore, it is not unusual to observe differential features of this area among fish species in relation to their different feed habits. For instance, in lepidophagous fishes, the occurrence of a large sub-terminal mouth coupled to a unique arrangement of teeth on the jaws enables this species to perform its specialized feeding behaviour, consisting of scraping and eating the scales of other fish [7,8,9,10,11,12,13,14,15,16,17,18]. Additionally in the headgut, it is possible to find other distinctive anatomical structures such as the pharyngeal jaw apparatus that, combined with the oral jaws, allows some fish species to crush shells, mince feed, and separate edible material from unwanted debris, thus expanding their feeding capabilities [19].

The first part of the foregut is characterised by the oesophagus, with a squamous epithelium in the anterior part and a columnar one in the posterior part [20]. Noteworthy, the epithelium of the foregut has a different origin from that of the midgut, namely ectodermal for the foregut and endodermal for the midgut [21]. In addition, the foregut is characterized by the presence of goblet cells that, by producing mucous, facilitate the acquisition of food, preventing abrasion as well as invasion of pathogens [22]. These cells contribute to form the mucous layer, beneath which, a submucosal layer composed of a thick mass of loose connective tissue combined with a muscular layer is immediately arranged, which is crucial for the integrity of the oesophagus wall [23]. The occurrence of a muscular layer, which is also coupled with long branched folds in some species, suggests a certain elasticity and stretching capacity of the luminal surface area of the oesophagus, particularly in predatory fish [20,24]. Furthermore, in some fish species, the oesophagus is characterized by the presence, especially in the cranial portion, of several taste buds that are helpful for food selection [25].

Following the oesophagus, as in other vertebrates, it is quite common to recognise the stomach. Nevertheless, it is worthy to highlight that the stomach is lacking in 20% of fish species (i.e., Gobiidae, Blennidae, Cyprinadae), where it is partly replaced by other adaptations such as well-developed pharyngeal teeth, pharyngeal pockets, secretory glands present in the oesophagus, or a muscular gizzard [26,27]. In fish species where the stomach occurs, it may assume different conformations (straight, U-shaped, or Y-shaped), normally linked to the feeding habits of the specimens. For instance, the U-shaped stomach is typical of herbivores/omnivores, whereas the Y-shaped stomach is present in some predators since it allows the storage of large amounts of food [7,28]. Despite the different morphological structures, it is possible to recognize three distinct regions in fish stomach, defined as cardiac, fundic, and pyloric [17]. The primary roles of the fish stomach are the storage of food and the production of hydrochloric acid (HCl), which is necessary to promote the digestion process. The HCl secreted by the parietal cells of gastric glands generates the strongly acidic environment (pH < 2) of the gastric lumen, which is crucial for the correct activity of several digestive enzymes [21,22]. The gastric gland allocation appears to be greater in the anterior than in the posterior part of the stomach, the latter represented by the pyloric region that performs a key role in food storage rather than digestion. The occurrence of neutral mucins throughout the stomach, produced by the columnar epithelial cells, ensures a dual protective function against the presence of microorganisms and the high acidity of the gastric lumen [17,24,28]. Even though digestion into the stomach is primarily carried out by digestive enzymes, in some fish species (i.e., Clupeoidei, Channidae, Mugilidae, Acipenseridae, Coregoninae, and Chanidae), it is possible to notice a pyloric stomach able to grind food, acting as a sort of “gizzard” [5,21].

In the midgut, the forward part of the gut, the assimilation of digested food into the stomach begins. In order to fulfil this task, the pyloric caeca are located in this portion. The pyloric caeca are finger-like outgrowths whose shape permits an increase of the absorption surface area without promoting fermentation and storage, as demonstrated by [29]. Similar to the other portions of the GIT, differences in regard to size, state of branching, and connection to the gut are detectable in the various fish species. For instance, in the Mugilidae, the number of pyloric caeca is a crucial element for the identification of the species [30]. However, no clear correlation seems to exist between the pyloric caeca shape and feed habits of fish [31,32]. Interestingly, the midgut is the longest portion of the gut and its length is extremely variable and generally correlated with the feeding habits of the fish species. For instance, carnivorous species frequently possess intestines that are shorter than herbivorous fish, with large coils occurring in the former [33]. From a histological perspective, three layers, namely the mucosa, muscularis externa, and serosa, may be observed in the gut [26]. In the mucosa layer, the columnar epithelium plays the absorptive role, as testified by the presence of a distinctive brush border in the apical part. In the same layer, it is possible to observe other types of cells such as goblet-type mucous cells, lymphocytes, enteroendocrine cells, and rodlet cells [34], each one performing a different role. Indeed, the goblet cells are mucous cells [21], the enteroendocrine cells produce neuroendocrine substances [35], and the lymphocytes and rodlet cells have an immune function [36,37]. In contrast to mammals, in the fish gut, it is not possible to identify the villi organisation, but rather the existence of irregular random folds without crypts. Lieberkuhn crypt-like structures are encountered only in the Gadidae, such as cods [21,26].

The terminal part of the GIT is the hindgut. The hindgut of most fish is short and very difficult to distinguish from the midgut in terms of histomorphology, in particular with respect to changes in diameter or epithelial morphology. Additionally, in the hindgut, unlike the midgut, it is possible to observe an increased level of mucus production [5,32] and variations with regards to the mucosal fold and muscle thickness [21]. In the posterior intestine, unlike the midgut, a higher amount of goblet cells may be observed, resulting in greater mucous production. The rise in the presence of goblet cells from the anterior to posterior intestine, common in various fish species, provides epithelium protection and lubrification in order to facilitate food flow and defecation [17,22,38,39].

## 3. The Interaction between Fish Immune System and Microbiota

As previously described, a mucous layer is present, lining the entire lumen of the GIT. This mucous system allows for the development of an immune system, able to enhance innate or adaptive immune responses [40]. The immune system avoids the invasion of opportunistic and pathogenic microorganisms, whilst simultaneously preserving and enhancing the proliferation of commensal bacteria [41,42,43]. The innate immunity represents the main defence line for fish, despite the occurrence of an adaptive immune system, which has a restricted antibody repertoire and slower lymphocyte maturation compared to mammals [44]. The innate immunity is composed of physical barriers, humoral, and cellular components [40]. The physical barriers are characterized by a series of mucosal structures located in the skin, gills, and gut, which are able to prevent pathogen invasion. In the mucosal secretions, it is possible to observe several molecules such as:

- Antimicrobial peptides (AMPs), which are small peptides (up to 50 amino acids residues). In fish species, it is possible to find a variety of major groups of AMPs such as the piscidins, cathelicidins, defensins, hepcidins, and high-density lipoproteins [44]. AMPs can guarantee general microbiota homeostasis thanks to their antimicrobial activity of immunity modulation [45].
- Lysozymes, which are other useful components against pathogenic microorganisms. Indeed, these enzymes are able to cleave the glycosidic bond between the N-acetylmuramic acid and N-acetylglucosamine of bacterial cell wall peptidoglycans [46]. In fish, the role of lysozymes against pathogens has been demonstrated in several studies [47,48,49,50], which reported the modulation in their expression and activity versus several fish pathogen bacteria, such as *Streptococcus iniae* or *Vibrio alginolyticus*.
- Complement system, which represents the element able to connect the innate and adaptive immunity. Approximately 30 inactive circulating proteins and membrane-bound receptors belong to this system [51]. The activation of these proteins is related to three different pathways: the classical pathway, where antigen-antibody complexes act as activators; the alternative pathway, where the activation is caused by the presence of several molecules on the surface of microorganisms without the antibodies attendance; and the lectin pathway, where the activation occurs after the mannose-binding lectin binds to the cell-surface carbohydrates of microorganisms [52,53]. Regardless of the three complement pathways, the C3 protein always plays the main role in the function of the complement system. Indeed, the C3 cleavage of C3 generates two protein subunits, namely C3a and C3b. C3b is fundamental because it activates the lytic pathway by attaching itself to the pathogen surface. In addition, C3b permits the sequential connection of C5b, C6, C7, C8, and C9 proteins to form the membrane-attack complex (MAC), able to provoke cell lysis [53].
- Other useful compounds, such as transferrin, pentraxins, and lectins. These compounds represent further useful weapons to avoid the growth of dangerous bacteria and allow for beneficial microbiota homeostasis [43,44,51].

Among the physical barriers and antimicrobial compounds, several kinds of cells, such as the monocytes/macrophages, neutrophils, dendritic cells, and natural killer cells, represent the cellular component of the innate immune system in fish species. These cells are present in the Gut-Associated Lymphoid Tissue (GALT), where it is also possible to observe the lymphocytes, fundamental in the adaptive immune system. In contrast to mammals, in the fish gut, these cells are located between the epithelium and lamina propria, without a tissue organization [43]. Additionally, the epithelium is able to corroborate to the immune responses, producing specific receptors called Toll-like receptors (TLRs), which are important mediators of inflammatory pathways in the gut, playing a key role to recognize specific microbial patterns [54]. When this pattern is linked to the receptor, the cells start to produce several signal molecules (i.e., tumor necrosis factors (TNF), interleukins, and chemokines) able to guarantee the recruitment of innate immune cells. The innate immune cells (i.e., monocytes/macrophages, neutrophils, dendritic cells, and natural killer cells) use differential mechanisms of action to inhibit microbial proliferation. Indeed, in order to destroy pathogenic organisms, the innate immunity involves the activation of phagocytose (mediated by monocytes/macrophages) and the release of cytotoxic molecules (mediated by natural killer cells) or cytokines for the recruitment of adaptive immune cells [51]. For several years, researchers had supposed that adaptive immune cells did not have a specific role in the microbiota balance; however, more recent studies [55,56] suggested a possible implication in this context, mediated mainly by lymphocytes [57]. Although the role of the B and T lymphocytes seems to be not so different, some studies indicated that T and Z immunoglobulins (Ig) produced by the B lymphocyte are able to guarantee correct microbial colonization [43,58]. It has been reported that IgT is the primary immunoglobulin produced in response to the pathogenic microbiome in the fish gut and prevents any damaging effects of pathogens on the fish [3]. In general, the presence of specific bacteria is required for normal gut development in terms of the proliferation of epithelial cells and maturation of the immune system [43]. As a matter of fact, studies of the gnotobiotic zebrafish have pointed out the relevance of the microbiota in the proliferation of the intestinal epithelial cells by involving MyD88 [59] and in promoting the movement of immune cells to the intestine, such as the migration of neutrophils induced by commensal microorganisms activating serum amyloid A [60]. Additionally, the microbiota is crucial for regulating the neutrophil activity by enhancing the expression of several pro-inflammatory genes [61,62], thus improving infection resistance [43].

## 4. The Interaction between Fish GIT and Microbiota

The microorganisms that belong to fish microbiota can colonize several parts of the fish body such as the skin, gills, and obviously the gastrointestinal tract. Indeed, although the gut, with its morphology, helps the microbial colonization, other portions of the fish GIT can host different microorganisms, such as bacteria, fungi, and viruses [7,63]. Bacteria represent the most abundant component throughout the fish GIT, with an amount ranging from 10^7^ to 10^11^ microorganisms per gram of intestinal content [2,12,64]. As a result, the majority of the research studies are primarily concerned with them. As in other animals, the microbiota colonization withstands the influence of different factors. Agents such as the environment (freshwater or saltwater), the feed habits (herbivores, carnivores, omnivores, or detritivores), and GIT shape are among the main ones [3]. The influence of the environment is confirmed by various studies [2,63] that highlighted how the genera *Alteromonas*, *Flavobacterium*, *Pseudomonas*, and *Vibrio* are more abundant in saltwater fish species, while in freshwater ones, the main microbial genera are represented by *Aeromonas*, *Lactococcus*, *Pseudomonas*, and *Clostridium*. A comparison of the main microorganisms found in the GIT of freshwater and saltwater fishes is reported in Table 1, together with data about the dominant bacteria stains typically detected in mammals.

As with other species, even among fish, it is possible to distinguish among different feed habits. The diet of a species is the result of specific adaptations that are also reflected in the digestive system, including the occurrence of digestive enzymes. Since a significant part of the digestive enzymes are synthesized by bacteria, the connection between the trophic level of a species and microbiota is not unusual [72]. Indeed, different studies [4,7,73] have documented the prevalence of some bacteria in fish species with similar feed habits [63], as also highlighted by [72], which reported the prevalence of cellulose-degrading bacteria such as *Clostridium*, *Citrobacter*, and *Leptotrichia* in the herbivores, while protease-producing bacteria, such as *Cetobacterium* and *Halomonas*, were reported in the carnivorous fish species. A more detailed list with the dominant microbial phyla found in the GIT of fish species of commercial relevance, grouped according to their feed habits, is reported in Table 2.

As described previously, in the fish GIT, is possible to recognize different regions, and each one is characterized by a different microbial density. Indeed, a certain amount of bacteria is detectable along the entire gastrointestinal tract, but some parts of the GIT are more colonized than others. In general, in fish, the bacterial colonization seems to follow an increasing trend from the stomach to the hindgut [63]. Nevertheless, this tendency is also associated with other different situations of constant microbial density along the fish GIT [95], or a reduction in the amount of microbiota from the mouth to the anus [96]. In addition to the previously reported quantitative differences, a wide range of microbial species can be identified in the various parts of the digestive tract in different fish species, as reported in Table 3.

Data reported in the previous published articles allow us to create a sort of “map” detailing one microbial species is more commonly present than another. The foregut, in particular the stomach, is not often subjected to microbiota analysis in comparison to the other regions of the fish GIT because it is thought that the data obtained can be easily affected by the fast movement of the food. Nevertheless, some research studies [7,97,98,99] have focused on the microbiota in the stomach, demonstrating the dominance of the phyla Firmicutes, Proteobacteria, and Actinobacteria in fish species such as *Sparus aurata* and *Epinephelus awoara*. These findings may suggest how these microbial phyla could likely adapt to both the low gastric pH and the activity of proteolytic enzymes [99].

In the other regions of the fish GIT, namely the midgut and hindgut, the physico-chemical conditions are more suitable for microbial proliferation than those observed in the foregut, particularly in stomach, and therefore, the bacteria present in the gut differ from those found in this area [105]. In certain cases, even the microbial population detected in the midgut and hindgut might differ, as different bacteria genera, such as *Pseudomonas* for the midgut and *Vibrio* for the hindgut, were, for instance, detected in *Salmo salar* and *Gadus morhua* [70,90]. Given the natural connection established between the host’s microbiota and its health status [43], the knowledge gained so far regarding the microbial communities offers useful information to optimize strategies of gut microbiota handling with the aim of enhancing the health status of fish, especially in the aquaculture sector [7].

## 5. Use of Feed Additives for Fish Health Improvement and Gut Efficiency

Over the last decades, the increased development of aquaculture activity has led to a growing attention on the wellness of fish species of commercial relevance. Nevertheless, the productivity growth of the aquaculture sector appears to be challenged by the frequent infections impacting the commercial fish species triggered by pathogens. Among the numerous forms of stressors, bacterial infections are one of the most widespread causes of harm in fish farming, with considerable economic implications [106]. To counteract this serious issue, as in other kinds of rearing, antibiotics are routinely used in the aquaculture sector. However, the extensive use of antibiotics to avoid the onset of diseases in aquaculture facilities may lead to the leakage of these drugs into wastewater, and consequently into rivers and seas [107,108]. Antibiotics, particularly those present in rivers, have the ability to permeate soil and enhance the selection and growth of antibiotic-resistant bacteria [109]. The variety of environmental implications notwithstanding, antibiotic usage can lead to a substantial loss of gut microbiota variability [110]. Generally, the conditions which fish are subjected to in fishery farms might be a cause of stress. Indeed, the continuous maintenance of different environmental factors such as temperature, photoperiod, and salinity in aquaculture plants is not always straightforward, and significant fluctuations in these parameters are a very common occurrence. As a result, the stress exposure in farmed fish may have a negative impact on a variety of physiological mechanisms, including the immune system [111]. Therefore, due to the huge impact that antibiotics have on the environment and health of farmed fish, it is crucial to establish alternative solutions capable of preserving the well being of farmed species and at the same time, reducing the ecological harm [112].

According to numerous recent studies [13,111,112,113,114], feed additives such as prebiotics and probiotics provide a tool capable of boosting aquaculture plant productivity by acting on a variety of ecological and biological aspects while minimizing the antibiotic consumption. Besides being identified as a very good alternative to the usage of antibiotics, the use of these feed additives in aquaculture represents a useful strategy for improving the overall performance of the aquaculture sector since it affects a variety of factors including rearing water quality [115,116], food absorption, and digestion, and has shown a positive effect on the growth performance of fish [117,118,119]. From an immunological perspective, the activity of these feed additives does not merely promote the proliferation of commensal bacteria at the detriment of pathogens. In actuality, it has been demonstrated that prebiotics and probiotics may enhance and improve the immune response of fish species by either increasing the leukocyte amount or the activity of key proteins such as lysozymes, as well as the complement system [120,121,122,123]. More in detail, data from the literature on the administration of specific prebiotics (Table 4) and probiotics (Table 5) acting as effective immunostimulants on aquaculture fish species from seawater and freshwater are listed in the following tables.

Ensuring the health of aquaculture species has an impact on parameters such as nutrient uptake and the growth performance of fish. Growth performance improvement is another relevant target for dietary supplements, since it can lead to a higher output, and hence to higher earnings for the fishery plant. The purpose of new feed formulations is, therefore, to assure an optimum feed absorption and performance of fish growth whilst reducing the rearing costs. In many cases, it seems fundamental to increase the digestive enzymes in order to maximize nutrient absorption. Indeed, numerous studies [117,156,219,220] have shown that the addition of prebiotics and probiotics enhances the nutrient absorption from feed, since they act on a variety of enzymes such as amylases and proteases [119]. However, in other cases, it was also observed that the new diet formulations could have a deleterious impact on the morphological structure of the fish gut, triggering some inflammatory responses [221]. This condition, especially if maintained for a prolonged period, might disrupt the architecture of the intestine, and therefore lead to the upset of the general health of the microbiota and the organism itself. To overcome this situation, the use of prebiotics and probiotics might be helpful. As a matter of fact, several studies [9,222,223] have demonstrated how the intake of prebiotics and probiotics may preserve and, in some cases, improve the morphology and the function of gut, often affected by the rearing conditions. More in detail, data from the literature on the administration of specific prebiotics (Table 6) and probiotics (Table 7) enhancing the gut efficiency and growth performance of aquaculture fish species from seawater and freshwater are listed in the following tables.

## 6. Use of Histomorphological Assays to Estimate the Quality of Feed Additives

As previously stated, the selection of new feed formulations plays an essential role in the well being of the specimens, and hence, the yield of the aquaculture activity [106]. Nevertheless, the formulation of optimal feeding diets for the wide variety of aquaculture fish species requires the implementation of different types of analyses to verify their impact on the health status of the specimens [251]. Among the different approaches employed [194,252], histomorphological assays represent a good biomarker for the assessment of the welfare of aquatic organisms [253,254,255,256,257] since it is able to promptly provide insights into the overall health status of individuals under examination. In particular, in the histological assays of fish guts, endpoints such as mucosal fold length (villi), muscle thickness (MT), and crypt depth (CD) might provide useful information regarding the efficiency of the gut in terms of nutrient absorption [223], while evaluation of the number of goblet cells and leukocytes may be useful for estimating the state of the immune system response, which is crucial for gut microbiota balance [222]. The evaluation of these endpoints may be performed by using both optical and electron microscopy, which offer useful information for an accurate assessment of the actions of prebiotics and probiotics at the fish-GIT-tissue level, as summarized in Table 8.

For instance, a number of histomorphological parameters were used to assess the impact of lactic acid bacteria and yeast on *Oreochromis niloticus* [222], including the haematoxylin and eosin (H/E) and periodic acid-Sciff (PAS) staining, which allowed for an observation of a relevant increase in the intestinal perimeter ratio and mucosal fold length (villi) after the probiotic treatment. These kinds of alterations in the fish gut structure may result in a facilitated and increased feed absorption and growth performance [264]. In parallel, the number of goblet cells and leukocytes in treated samples were found to be higher than those in control fish, highlighting the positive effect of the administrated microorganism on the immune system of fish. Similar results were obtained in *O. niloticus* challenged with the combined treatment of the probiotic *P. acidilactici MA18/5M* (Bactocell, Lallemand SAS, France) and the prebiotic short-chain fructo-oligosaccharides (FOS), usually termed as symbiotic [265], which enhanced the intestinal structure with increases in the mucosal fold (villi) height and the immune system with elevation of intraepithelial leukocytes, granulocytes, and goblet cells [258]. Notably, the combined administration of three probiotic strains (*Bacillus coagulans*, *B. licheniformis* and *P. polymyxa*) on the northern whiting *Sillago sihama Forsskål* induced positive effects in terms of gut wellness, resulting in higher uptake of intestinal nutrients and better growth performance, as demonstrated by an evaluation of some histological parameters (i.e., villus height and width, muscle thickness, and crypt depth) and growth and immune response endpoints [223].

The primary aim of these current research studies is to determine the optimal feed additives for each aquaculture species while also considering the dosage [266]. Indeed, the inappropriate usage of certain feed additives may have negative effects in certain circumstances, as reported in gilthead sea bream *Sparus aurata*, in which the combined intake of inulin and *B. subtilis* caused oedema and inflammations in the fish gut, as observed by transmission electron microscopy (TEM) [221]. Contrarily, Moroni et al. [9] documented in the same fish species that the probiotic *Lactococcus lactis* does not provoke any impairments in gut morphology nor inflammation. Therefore, these findings suggest the need to employ the most appropriate feed additive for each individual fish species. Interestingly, the supplementation of nucleotides in the diet of red drum *Sciaenops ocellatus* was capable of improving several histomorphological parameters in different gut tracts (distal intestine, mid-intestine, proximal intestine, pyloric caeca), enhancing the general health status of the fish and its performance in terms of nutrient absorption [230].

Feed additives can be therefore considered as a useful tool in the design of new food formulae for a more sustainable aquaculture. Indeed, the inclusion of the probiotics *Lactobacillus brevis* and *L. buchneri* in the diets of *Seriola dumerili*, in which fish oil was replaced by vegetable oils, reduced the thickness of submucosa layer of the posterior intestine, thus lowering inflammatory condition [259]. *Lactobacillus* spp., along with *Bacillus* spp., are probably among the most widely used probiotics in aquaculture [170,175,267,268]. Moreover, ref. [264] elucidated the ability of the probiotics *B. licheniformis* and *B. subtilis* coupled to the yeast *S. cerevisiae* to enhance several histological parameters such as the number of goblet and mucosal cells, and villus length and width. Interestingly, ref. [261] documented that although the individual use of the commercial probiotic Aquablend^®^ had a significant effect on fold length in the proximal section of the GIT in *Totoaba macdonaldi*, its use in combination with the prebiotic GroBiotic^®^-A showed an increase in the values of these parameters, though not significantly, as a signal that the formulation used needs to be revised.

To accurately integrate and interpret the histological results, the use of a continuous scale scoring system may be helpful, as applied and reported in [262] for the evaluation of a number of histological parameters (i.e., intestinal folds, changes in enterocytes nucleous, supranuclear absorptive vacuolisation, connective tissue hyperplasia in the lamina propria and submucosa, infiltration of inflammatory intraepithelial leukocytes), that allowed for an understanding of the inefficiency of prebiotics (FOS and xylo-oligosaccharides) to counteract the negative impact provoked by diets including plant feedstuffs in the distal intestine of juveniles *D. labrax*.

Besides enhancing the general wellness of aquaculture fish species, feed additives (prebiotics and probiotics) play a pivotal role in challenging infections, providing a valid alternative to the use of antibiotics that may lead to antibiotic resistance after a prolonged period of time [175,256,257,269]. In [175], the efficiency of three *Bacillus* species in *O. niloticus* against *A. hydrophila* infection was assessed, resulting in improvements in some histological parameters of the fish gut (i.e., villus length and width, goblet cells count, intestinal epithelial muscle thickness). Noteworthy, the rise in the number of goblet cells is relevant to challenging infection, as these cells secrete mucus-containing bactericidal compounds which are useful against pathogens [115]. Therefore, this histological parameter, combined with other endpoints such as mortality rate, could provide relevant information on the immunostimulant and pathogen resistance effects of the tested feed additives. A further relevant histological parameter to estimate the pathogen resistance effects of feed additives is the level of intraepithelial leukocytes, as reported by [172,263] after using the commercial probiotic AquaStar^®^ Growout (*L. reuteri*, *B. subtilis*, *E. faecium* and *P. acidilactici*) in tilapia *O. niloticus*. Finally, the evaluation of microvilli density by TEM, besides providing information on the absorptive surface area index (combined with numerical data of microvilli length and perimeter ratio), may also reveal tight junctions between enterocytes, and thus, the occurrence of a barrier against pathogens [263]. Therefore, these results point out the potential of the histological approach for obtaining a variety of precious information to delineate the gut health status of fish species treated with feed additives.

## 7. Conclusions

In this review, the close relationship between the microbiota and the GIT of fish is highlighted. The occurrence of different environmental conditions (freshwater and seawater) and dietary habits (herbivores, carnivores, omnivores, detritivores) are able to influence this ratio, resulting in changes both in terms of microbial colonization and histomorphological patterns, with repercussions in fish GIT efficiency. Therefore, the use of histological endpoints to assess the general health status of aquaculture fish species treated with feed additives (prebiotics and probiotics) represents a very useful strategy for understanding the capacity of these treatments to promote the interface between the microbiota and the gut, which is crucial for its proper performance in terms of nutrient uptake and defense against pathogens. Overall, an in-depth knowledge of the appropriate administration of feed supplements for individual fish species is a promising strategy to greatly enhance the aquaculture sector, both in terms of quality and sustainable production. Therefore, this review has the potential to provide directions for future research in this field.

## Figures and Tables

**Table 1:** Comparison of the main microorganisms found in the gastrointestinal tract (GIT) of mammals, freshwater, and saltwater fishes.

	Amount of Bacteria in GIT	Dominant Microbial Phyla	Less Abundant Microbial Phyla	References
**Mammals**	10^8^ microbial cells for gram of intestinal content	Bacteroidetes, Firmicutes	Proteobacteria, Actinobacteria, Fusobacteria, Cyanobacteria	[65,66,67]
**Freshwater fishes**	10^7^ to 10^11^ microbial cells for gram of intestinal content	Proteobacteria, Fusobacteria, Firmicutes, Bacteroidetes, Actinobacteria *Aeromonas*, *Pseudomonas*, *Bacteroides* type A	Verrucomicrobia, Enterobacteriaceae, *Micrococcus*, *Acinetobacter*, *Clostridium*, *Bacteroides* type B, *Fusarium*, *Plesiomonas*	[11,12,68,69,70,71]
**Saltwater fishes**	10^7^ to 10^11^ microbial cells for gram of intestinal content	Proteobacteria, Fusobacteria, Firmicutes, Bacteroidetes, Actinobacteria *Vibrio*, *Pseudomonas*, *Acinetobacter*, *Corynebacterium*, *Alteromonas*, *Flavobacterium*, *Micrococcus*	Verrucomicrobia	[11,12,68,69,70]

**Table 2:** Comparison of the main microorganisms found in the gastrointestinal tract of fish species according to their feed habits. (SW—seawater; FW—freshwater; AE—anadromous euryhaline).

Feed Habits	Fish Species	Dominant Microbial Phyla	References
Herbivores	*Acanthurus nigricans* SW	Bacteroidetes, Proteobacteria, Firmicutes	[74]
	*Acanthurus* sp. SW	*Epulopiscium*	[75]
	*Aplodactylus arctidens* SW	*Clostridium*, *Eubacterium desmolans*, *Papillibacter cinnaminovorans*	[76]
	*Chlorurus sordidus* SW	*Vibrio*, *Photobacterium*	[74]
	*Hermosilla azurea* SW	*Enterovibrio*, *Bacteroides*, *Faecalibacterium*, *Desulfovibrio*	[77]
	*Kyphosus* spp. SW	Gamma-proteobacteria, Clostridia, Bacteroidia, Erysipelotrichia (*Vibrionaceae*, *Pasteurellaceae*, *Erysipelotrichaceae*)	[78]
	*Odax pullus* SW	*Clostridium*	[76]
	*Ctenopharyngodon idellus* FW	*Clostridium*, *Citrobacter*, *Leptotrichia*	[72]
	*Megalobrama amblycephala * FW	*Brevundimonas*, *Massilia*, *Curvibacter*, *Acinetobacter*, *Clostridium*, *Citrobacter*, *Leptotrichia*	[72,79]
Carnivores	*Acipenser baerii* SW	*Cetobacterium*	[80]
	*Chaenocephalus aceratus* SW	*Photobacterium*	[81]
	*Cynoscion nebulosus* SW	*Escherichia*	[82]
	*Epinephelus coioides* SW	*Bacillus*, *Vibrio*, *Delftia*, *Psychroacter*, *Acinetobacter*, *Pseudomonas*	[83]
	*Fugu niphobles* SW	*Vibrio*, *Pseudomonas*, *Flavobacterium*	[84]
	*Gadus morhua* SW	*Clostridium*, *Vibrio*	[85]
	*Hippoglossus hippoglossus* SW	*Vibrionaceae* (in larval and juvenile stages), *Photobacterium phosphoreum* (in adults)	[71]
	*Lutjanusn bohar* SW	*Vibrio*, *Photobacterium*	[74]
	*Morone saxatilis* SW	*Aeromonas*, *Pseudomonas*, *Vibrio*	[86]
	*Notothenia coriiceps* SW	*Photobacterium*, *Vibrio*	[81]
	*Paralichthys lethostigma* SW	*Clostridium*, *Photobacterium*,	[82]
	*Pomatomus saltatrix* SW	*Vibrio*, *Pseudomonas*, *Enterobacteraceae*	[87]
	*Sciaenops ocellatus* SW	*Mycoplasmataceae*, *Photobacterium*, *Cetobacterium*, *Clostridiaceae*, *Vibrio*	[82]
	*Sparus aurata* SW	*Pseudomonas*	[88]
	*Salmo trutta trutta* SW	*Aeromonas*, *Pseudomonas*	[89]
	*Salmo salar * AE	*Acinetobacter junii*, *Mycoplasma*, *Lactobacillus*, *Photobacterium phosphoreum*, *Lactococcus*, *Bacillus*	[90]
	*Culter alburnus* FW	*Cetobacterium*, *Halomonas*	[72]
	*Pelteobagrus fulvidraco* FW	*Clostridium*, *Yersinia*, *Aeromonas*, *Clostridiales*	[79]
	*Siniperca chuatsi* FW	*Cetobacterium*, *Halomonas*	[72]
Onnivores	*Gillichthys mirabilis* SW	*Mycoplasma*	[91]
	*Lagodon rhomboides* SW	*Clostridium*, *Mycoplasma*, *Photobacterium*, *Propionibacterium*, *Staphylococcus*, *Pseudomonas*, *Corynebacterium*	[82]
	*Cariassus auratus* FW	*Brevundimonas*, *Massilia*, *Curvibacter*, *Delftia* *Clostridium*, *Cetobacterium Halomonas*	[72,79]
	*Cyprinus carpio* FW	*Brevundimonas*, *Massilia*, *Curvibacter*, *Sphingobacteriales*, *Cetobacterium*, *Aeromonas*, *Chitinophaga*, *Halomonas*, *Clostridium*	[72,79]
Zooplanktivores	*Apogonidae* SW	*Vibrionaceae*, *Pasteurellaceae*, *Vibrio harveyi*, *Shewanella* sp., *Endozoicomonas* sp.	[73]
	*Clupea harengis* SW	*Pseudomonas*, *Alteromonas*, *Psychrobacter*	[92]
	*Pomacentridae* SW	*Vibrionaceae*, *Pasteurellaceae*, *Vibrio harveyi*, *Shewanella* sp., *Endozoicomonas* sp.	[73]
	*Sardinella longiceps* SW	*Achromobacter*, *Vibrio*, *Pseudomonas*	[93]
	*Scomber scombrus* SW	*Psychrobacter*, *Vibrio*, *Shewanella*	[94]
	*Syngnathus scovelli * SW	*Proteobacteria*	[82]

**Table 3:** Distribution of microorganisms along the gastrointestinal tract (GIT) of different fish species. (SW—seawater; FW—freshwater; AE—anadromous euryhaline).

GIT	Fish Species	Dominant Phyla	References
Stomach	*Sparus aurata* SW	Firmicutes, Proteobacteria, Bacteroidetes (*Bacillales*, *Flavobacteriaceae*)	[97]
	*Sparus aurata* SW	Firmicutes, Proteobacteria, Actinobacteria	[98]
	*Epinephelus awoara* SW	Firmicutes, Proteobacteria, Actinobacteria, *Deinococcus-Thermus*, *Planctomycete*	[99]
	*Scophthalmus maximus* SW	Proteobacteria, Firmicutes, Bacteroidetes, Tenericutes, Actinobacteria, Cyanobacteria	[100]
	*Oncorhynchus mykiss* FW	Proteobacteria, Firmicutes, Fusobacteria *(Cetobacterium dominanti)*	[101]
Foregut (oesophagus, stomach, pylorus)	*Salmo salar* AE *Gadus morhua* SW	Proteobacteria (*Janthinobacterium*, *Pseudomonas*, *Acinetobacter*, *Vibrio)*	[70,90]
	*Scophthalmus maximus* SW	Proteobacteria, Firmicutes, Tenericutes, Actinobacteria, Cyanobacteria	[100]
Midgut	*Salmo salar* AE *Gadus morhua* SW	Proteobacteria *(Photobacterium phosphoreum*, *Pseudomonas)*	[70,90]
	*Pelteobagrus* *fulvidraco* FW	Firmicutes, Proteobacteria, Bacteriodetes, Fusobacteria	[102]
	*Oncorhynchus mykiss* FW	Proteobacteria, Firmicutes, Fusobacteria, *Bacteroidetes* (*Bacillus*)	[101]
	*Ctenopharyngodon idella* FW	Proteobacteria, Firmicutes, Actinobacteria (*Anoxybacillus*, *Leuconostoc*, *Clostridium*, *Actinomyces*, *Citrobacter*)	[103]
Hindgut	*Salmo salar* AE *Gadus morhua* SW	*Vibrio*, *P. phosphoreum*	[70,90]
	*Scophthalmus maximus * SW	Proteobacteria, Firmicutes, Tenericutes	[100]
	*Acipenser baerii * AE	Fusobacteria/Firmicutes, Chlamydiae, Bacteriodetes, Actinobacteria	[80]
	*Danio rerio* FW	Firmicutes, Proteobatceria, Bacteriodetes	[104]

**Table 4:** Administration of prebiotics acting as effective immunostimulants in different aquaculture fish species. (SW—seawater; FW—freshwater; AE—anadromous euryhaline; MOS—mannan-oligosaccharides; FOS—fructo-oligosaccharides; COS—chito-oligosaccharides; GOS—galacto-oligosaccharides).

Prebiotics	Fish Species	Dosage and Timing of Administration	Initial Weight (g)	Effects	References
Arabinoxylan-oligosaccharides	*Acipenser baerii * AE	2% for 28 days and 12 weeks	20.00	Increase of phagocytic activity of leukocytes	[124]
β-glucan	*Epinephelus coioides* SW	1, 2 g/kg for 30 days	6.40 ± 0.65	Enhancing of innate immune responses	[125]
β-glucan + MOS	*Cyprinus carpio * FW	Immunogen^®^ at 0.5, 1, 1.5, 2.5 g/kg for 8 weeks	11.12 ± 0.55	Increase of leukocyte count and resistance to *Aereomonas hydrophila*	[126]
COS	*Cyprinus carpio * FW	0.2% for 8 weeks	24.90 ± 0.52	Increase of lysozyme level	[127]
COS	*Scophthalmus maximus * SW	75, 150, 300, 600, 1200 mg/kg for 56 days	2.10 ± 0.10	Increase of phagocytic activity at doses > 300 mg/kg	[128]
COS	*Scophthalmus maximus * SW	2 g/kg for 56 days	11.00	Reduction of gene expression of pro-inflammatory cytokines	[129]
FOS	*Atractosteus tropicus* SW (Larvae)	7.5 g/kg for 15 days	0.03 ± 0.006	No significant increase in gene expression of pro-inflammatory cytokines	[130]
FOS	*Megalobrama terminalis * FW	0.3, 0.6% for 8 weeks	30.50 ± 0.50	Increase of various factors of immune system (IgM and complement proteins)	[131]
FOS	*Oreochromis niloticus * FW	0.5, 1, 2, 4 g/kg for 8 weeks	5.00 ± 0.02 g	Enhancing of immune and antioxidant responses, and resistance to *A. hydrophil*a (optimum at 1 g/kg)	[132]
FOS	*Salmo salar * AE	1% for 4 months	200.20 ± 0.60	No changes on immune system parameters	[133]
FOS	*Rutilus rutilus* FW	1%, 2%, 3% for 7 weeks	0.67 ± 0.03	Increase of immunoglobulins and lysozyme activity	[118]
FOS	*Paralichthys olivaceus * SW	0.5% for 56 days	21.00	Increase of lysozyme activity	[134]
FOS	*Acipenser stellatus* AE	1%, 2% for 11 weeks	30.16 ± 0.14	Increase of lysozyme activity at 1% dose	[135]
FOS	*Trachinotus ovatus* SW	2, 4 g/kg for 56 days	10.32	Increase of lysozyme activity and immunoglobulins	[136]
GOS	*Danio rerio* FW	0.5%, 1%, 2% for 56 days	0.045 ± 0.001	Enhancement of immune system parameters	[137]
Inulin	*Cyprinus carpio* FW	1, 2 g/kg for 60 days	25.37 ± 0.22	Enhancement of immunoglobulins of epithelial mucosa (optimum at 2 g/kg)	[138]
Inulin	*Huso huso * AE (juveniles)	1%, 2%, 3% for 8 weeks	16.14 ± 0.38	Increase of leukocyte count	[139]
Inulin	*Sparus orata * SW	10 g/kg for 2 and 4 weeks	50.00	Increase of immunglobulins IgM and immune responses, resistance to *P. damselae*	[140]
Inulin	*Oreochromis niloticus * FW	5 g/kg for 1 and 2 months	11.00	Increase of di haematocrit level, lysozyme activity and resistance to *A. hydrophila*	[120]
Inulin	*Lates calcarifer * FW	15, 20 g/kg for 60 days	7.14 ± 0.05	Enhancement of immune and blood parameters	[141]
Levan	*Cyprinus carpio * FW	0.1%, 0.2%, 0.5% for 75 days	9.00 ± 0.50	Increase of lysozyme level (optimum at 0.5% dose)	[142]
Levan	*Cyprinus carpio* FW	0.75% for 60 days	3.31 ± 0.52	Increase of myeloperoxidases and immunoglobulins	[143]
Levan	*Labeo rohita* *rohu* FW	0.1%, 0.25%, 0.50%, 0.75%, 1%, 1.25% for 60 days	4.50 ± 0.14	Increase of lysozyme level (optimum at 1.25% dose)	[144]
Levan	*Epinephelus coioides * SW	5, 10, 25, 50 g/kg for 12 weeks	6.00	Increase of lysozyme level (optimum at 25 g/kg)	[145]
MOS	*Salmo salar* AE	10 g/kg for 4 months	200.20 ± 0.60	Reduction of lysozyme activity	[133]
MOS	*Dicentrarchus labrax * SW	2%, 4% for 8 weeks	116.00	Increase of leukocyte activity and resistance to *Vibrio anguillarum* at 4% dose	[123]
MOS	*Oncorhynchus mykiss * FW	2 g/kg for 42 days	30.00	Increase of antibody titers and lysozyme	[146]
MOS	*Oncorhynchus mykiss * FW	0.4% for 12 weeks	13.20	Increase of phagocytic activity, haematocrit and resistance to *Vibrio anguillarum*	[147]
MOS	*Oncorhynchus mykiss * FW	0.25%, 0.5% for 12 weeks	36.27 ± 0.42	Increase of phagocytic activity, haematocrit and resistance to *Aereomonas salmonicida* at 0.5% dose	[148]
MOS	*Clarias gariepinus * FW	10 g/kg for 45 days	35.00	Increase of lysozyme activity	[149]
MOS	*Channa striata * FW	2 g/Kg for 12 weeks	10.00	Increase of lysozyme activity	[150]
MOS	*Pangasianodon hypophthalmus* FW	0.2%, 0.4%, 0.6%, 0.8% for 12 weeks	20.41 ± 1.64	Enhancement of immune parameters (immunoglobulins, lysozyme, leukocyte count) and resistance to *A. hydrophil*a	[151]
MOS	*Sciaenops ocellatus * FW	10 g/kg for 6–8 weeks	10.00	Increase of lysozyme activity	[152]
MOS	*Gadus morhua * SW	1 g/kg for 5 weeks	90.00	Increase of expression of interleukins and resistance to *V. anguillarum*	[153]
Nucleotides (NucleoforceFish™)	*Sparus aurata * SW	250, 500 mg/kg for 150 days	0.36 ± 0.002	Increase of gene expression of interleukins, hepcidin and B receptors of T cells	[154]
Sodium alginate	*Epinephelus coioides* SW	10 g/kg for 12 days	19.50 ± 0.50	Increase of immune responses (complement system, phagocytic activity, lysozyme)	[155]
Stachyose	*Scophthalmus maximus * SW	1.25%, 5% for 12 weeks	4.63 ± 0.01	Inhibition of microbial pathogen growth and increase of beneficial ones (optimum at 5% dose)	[156]
Thymol and carvacrol	*Sparus aurata * SW	0.01% for 9 weeks	26.00 ± 1.00	Increase of interleukins, cytokines and inflammatory responses	[157]
Thymol, carvacrol and essential oils from *Allium* spp.	*Sparus aurata * SW	0.5%	40.30 ± 0.1	Increase of immune innate responses at epithelial level and cortisol-mediated response, and lower microbial growth	[158]

**Table 5:** Administration of probiotics acting as effective immunostimulants in different aquaculture fish species. (SW—seawater; FW—freshwater; AE—anadromous euryhaline; CFU—colony-forming unit).

Probiotics	Fish Species	Dosage and Timing of Administration	Initial Weight (g)	Effects	References
*Aspergillus oryzae*	*Oreochromis niloticus * FW	1 × 10^10^ CFU/g	19.50 ± 0.50	Increase of non-specific immune and antioxidant responses	[159]
*Bacillus aerophilus*	*Labeo rohita * FW	1 × 10^6^, 1 × 10^7^, 1 × 10^8^, 1 × 10^9^ CFU/g for 6 weeks	35.00–40.00	Increase of lysozyme and phagocytic activity, and resistance to *A. hydrophila * (at 1 × 10^8^ CFU/g)	[160]
*Bacillus amyloliquefaciens*	*Labeo rohita* FW	1 × 10^5^, 1 × 10^7^, 1 × 10^9^ CFU/g for 70 days	20.23	Increase of antibody titers	[161]
*Bacillus amyloliquefacien*, *Bacillus pumilus*	*Pangasianodon Hypophthalmus* FW	1 × 10^8^, 3 × 10^8^, 5 × 10^8^ CFU/g for 90 days	15.30 ± 1.20	Increase of lysozyme and phagocytic activity, and resistance to *E. ictaluri* with reduced death rate (optimum at 5 × 10^8^ CFU/g)	[162]
*Bacillus licheniformis*	*Cyprinus carpio * FW	1 × 10^6^, 1 × 10^7^, 1 × 10^8^ CFU/g for 60 days	38.58 ± 0.42	Increase of pro-and anti-inflammatory citokines and resistance to *A. hydrophila* (optimum at 1 × 10^8^ CFU/g)	[163]
*Bacillus licheniformis*	*Oreochromis mossambicus* FW	1 × 10^5^, 1 × 10^7^ CFU/g for 4 weeks	24.00 ± 2.50	Increase of lysozyme activity and resistance to a *A. hydrophila*	[164]
*Bacillus licheniformis*, *Bacillus amyloliquefaciens*	*Centropomus undecimalis* SW (Larvae)	1 × 10^13^ CFU/g for 28 days		Reduction of lysozyme activity and not significant increase of SOD	[165]
*Bacillus licheniformis*, *Bacillus pumilus*	*Labeo rohita * FW	1 × 10^8^ CFU/g for 14 days	50.00–60.00	Increase of immune response and resistance to *A. hydrophila*	[122]
*Bacillus licheniformi*, *Bacillus subtilis*	*Ctenopharyngodon idella* FW	1 × 10^8^ CFU/g for 4 weeks	45.00	General enhancement of immune parameters	[166]
*Bacillus licheniformis Dahb1*	*Pangasius hypophthalmus* *FW*	1 × 10^5^, 1 × 10^7^ CFU/mL for 24 days	15.00 ± 2.50	Increase of lysozyme activity and myeloperoxidase (optimum at 1 × 10^5^ CFU/mL)	[167]
*Bacillus methylotrophicus*, *Bacillus amyloliquefaciens*, *Bacillus licheniformis*	*Labeo rohita * FW	1 × 10^7^ cells/g for 60 days	~50.00	Increase of immune and blood parameters, and resistance to *A. hydrophila*	[168]
*Bacillus pumilus*	*Oreochromis niloticus * FW	1 × 10^6^, 1 × 10^7^, 1 × 10^8^, 1 × 10^9^ CFU/kg for 30 days and 4 months	50.00	Increase of phagocytic activity and resistance to *Streptococcus agalactiae* (at 1 × 10^8^, 1 × 10^9^ CFU/kg)	[169]
*Bacillus pumilus A97*	*Trachinotus ovatus * SW	1 × 10^8^ CFU/g for 56 days	5.95 ± 1.69	Increase of expression of genes of non-specific immune responses (Toll-like receptor) and resistance to *Vibrio ponticus*	[170]
*Bacillus pumilus*, *Bacillus clausii*	*Epinephelus coioides* SW (Larvae)	1 × 10^6^ bacteria/mL for 28 days		Increased resistance to *Aliivibrio fischeri*, *Vibrio* *scophthalmi* and *Vibrio* sp.	[171]
*Bacillus* spp., *Enterococcus* spp., *Lactobacillus* spp. (AquaStar^®^)	*Oreochromis niloticus * FW	3 g/kg for 6 weeks	29.02 ± 0.33	Increase of expression of genes of immune responses (interleukins and cytokines), intraepithelial leukocytes and intestinal mucous cells	[172]
*Bacillus subtilis*	*Oncorhynchus mykiss * FW	1 × 10^4^, 1 × 10^5^, 1 × 10^6^, 1 × 10^7^, 1 × 10^8^, 1 × 10^9^ CFU/g for 14 days	30.00	Increase of leukocytes, lysozyme activity and resistance to *Aeromonas* sp. (optimum at 1 × 10^7^ CFU/g)	[121]
*Bacillus subtilis*	*Anguilla japonica * FW	1 × 10^6^, 1 × 10^7^, 1 × 10^8^ CFU/g for 8 weeks	8.29 ± 0.06	Increase of lysozyme activity and IgM (optimum at 1 × 10^7^ CFU/g)	[173]
*Bacillus thuringiensis*, *Bacillus cereus*	*Lates calcarifer* FW	1 × 10^12^ CFU/kg for 35 days	75.00 ± 0.60	Enhancement of antioxidant response resistance to pathogens (*V. harvey*)	[174]
*Bacillus velezensis TPS3N*, *Bacillus subtilis TPS*, *Bacillus amyloliquefaciens TPS1*	*Oreochromis niloticus* *FW*	1 × 10^8^ CFU/mL for 4 weeks	46.24 ± 0.48	Increase of lysozyme activity, IgM at epithelial mucosa and intestine, survival rate to *A. hydrophila*	[175]
*Bacillus amyloliquefaciens*	*Oreochromis niloticus* FW	2 × 10^6^ CFU/g for 60 days	1.49 ± 0.15	Enhancement of immune parameters and resistance to *A. hydrophila*	[176]
*Bacillus cereus*, *Bacillus subtilis*	*Oreochromis niloticus* FW	0.5 × 10^8^, 1 × 10^8^ CFU/g for 90 days	0.20 ± 0.05	Enhancement of immune status and resistance to diseases (optimum at 1 × 10^8^ CFU/g)	[177]
*Bacillus circulans*	*Catla catla * SW	2 × 10^4^, 2 × 10^5^, 2 × 10^6^ CFU/100 g for 60 days	6.48 ± 0.04	Enhancement of non-specific immune parameters (optimum at 2 × 10^5^ CFU/100 g)	[178]
*Bacillus pumilus*, *Bacillus clausii*	*Epinephelus coioides* SW (Larvae)	2 × 10^6^ bacetria/mL for 28 days		Increased resistance to *Aliivibrio fischeri*, *Vibrio scophthalmi*, and *Vibrio* sp.	[171]
*Bacillus* spp. *+ Lactobacillus* spp. *+ S. cerevisiae*	*Paralichthys olivaceus * SW	1 × 10^8^, 1 × 10^9^ CFU/kg for 12 weeks	13.50 ± 0.01	Enhancement of immune parameters	[179]
*Bacillus subtilis*	*Oreochromis niloticus * FW	0, 1, 2, 3, 4 g (1.19 × 10^8^ CFU/g)/kg for 50 days	14.82 ± 0.42	Enhancement of immune responses (optimum at 3 g/kg)	[180]
*Bacillus subtilis*	*Oreochromis niloticus * FW	1.1 × 10^5^ CFU/g for 84 days	5.26 ± 0.06	Enhancement of blood parameters (haematocrit and leukocytes)	[181]
*Bacillus aerius*	*Pangasius bocourti * FW	1 × 10^7^ CFU/g for 60 days	69.00	Increase of phagocytic activity, lysozyme, complement, and resistance to *A. hydrophila*	[182]
*Debaryomyces hansenii BCS004*	*Sparus aurata* SW	10^6^ CFUg^−1^ for 4 weeks	80.00 ± 5.00	Enhancement of immune parameters (phagocytic activity, increase of IgM)	[183]
*Enterococcus casseliflavus* *EC-001*	*Cyprinus carpio* FW	1 × 10^10^, 1 × 10^11^, 1 × 10^12^ CFU/kg for 56 days	12.00 ± 0.50	Increase of immune response and resistance to infections (optimum at 1 × 10^12^ CFU/kg)	[184]
*Enterococcus faecalis*	*Oncorhynchus mykiss * FW	5 × 10^8^ CFU/g for 30 days	50.00	Increase of resistance to *L. garvieae CECT 527*	[185]
*Enterococcus faecium*	*Rutilus rutilus caspicus * AE	1 × 10^7^, 1 × 10^8^ CFU/g	12.00	Increase of immunoglobulins and lysozyme activity	[186]
*Enterococcus faecium*	*Oreochromis niloticus* FW	1 × 10^7^ CFU/mL for 40 days	6.83 ± 0.18	Enhancement of immune parameters	[116]
*L. delbrueckii* sp. *delbrueckii AS13B*	*Dicentrarchus labrax * *SW* (Larvae)	1 × 10^5^ bacteria/cm^3^ for 74 days		Increase of T cells and granulocytes of intestinal mucosa	[187]
*Lactobacillus plantarum* (alone); *Bacillus subtilis*, *L. plantarum*, *L. rhamnosus*, *L. acidophilus*, *L. delbrueckii* (in mixture)	*Oreochromis niloticus* FW	1 × 10^8^ CFU/g for 112 days (by biofloc system)	8.63 ± 3.35	Increase of immune and antioxidant response, and resistance to pathogens (*A. hydrophila)*	[188]
*Lactobacillus rhamnosus ATCC 7469*	*Oncorhynchus mykiss* FW	1 × 10^9^ CFU/kg for 60 days	18.41 ± 0.32	Enhancement of non-specific immune parameters, gene expression of IL-1 and TNF, antioxidant activity and resistance to pathogens (*Yersinia ruckeri)*	[189]
*Lactobacillus acidophilus*	*Xiphophorus helleri * FW	1.5 × 10^8^, 3 × 10^8^, 6 × 10^8^ CFU/g for 70 days	0.03 ± 0.001	Increase of gene expression of immune responses (optimum at 6 × 10^8^ CFU/g)	[190]
*Lactobacillus acidophilus*	*Clarias gariepinus* FW	3 × 10^7^ CFU/g for 21 days	5.31 ± 0.10	Enhancement of blood parameters	[191]
*Lactobacillus acidophilus*	*Cyprinus carpio * FW	1 × 10^2^, 1 × 10^4^, 1 × 10^6^ CFU/kg for 56 days	21.34 ± 1.85	Increase of innate immune and antioxidant responses (optimum at 1 × 10^6^ CFU/kg)	[192]
*Lactobacillus delbrueckii*	*Cyprinus Carpio * FW	1 × 10^6^, 1 × 10^7^ CFU/g for 8 weeks	1.05 ± 0.03	Enhancement of intestinal immune parameters and resistance to *A. hydrophila*	[193]
*Lactobacillus fermentum*	*Cyprinus carpio * FW	1 × 10^8^ CFU/g for 56 days	3.90 ± 0.20	Enhancement of immune responses and resistance to pathologies	[194]
*Lactobacillus plantarum*	*Oreochromis niloticus * FW	3.4 × 10^8^, 6.8 × 10^8^, 1.3 × 10^9^ CFU/g for 40 days	24.50	Increase of gene expression of interleukins and cytokines (optimum at 6.8 × 10^8^ CFU/g)	[195]
*Lactobacillus plantarum*	*Oreochromis niloticus * FW	1 × 10^8^ CFU/g for 12 weeks	4.90 ± 0.04	Increase of lysozyme level in serum and epithelial mucosa, phagocytic activity and resistance to *Streptococcus agalctiae*	[112]
*Lactobacillus plantarum*	*Oreochromis niloticus* FW	1 × 10^8^ CFU/g for 84 days	5.92 ± 0.08	Increase of immune response in serum and epithelial mucosa	[196]
*Lactobacillus plantarum*, *Bacillus velezensis H3.1*	*Oreochromis niloticus * FW	1 × 10^7^ CFU/g *Bacillus velezensis*; 1 × 10^8^ CFU/g *Lactobacillus plantarum* for 30 days	21.80 ± 0.03	Increase of lysozyme and phagocytic activities, complement proteins and resistance to *Streptococcus agalactiae*	[197]
*Lactobacillus rhamnosus JCM1136*, *Lactococcus lactis* subsp. *lactis JCM5805*	*Oreochromis niloticus * FW (Juveniles)	1 × 10^8^ CFU/g for 6 weeks	0.20 ± 0.05	Increase of gene expression of immune response (interleukins, interferons) and survival rate	[198]
*Lactobacillus* spp., *Bacillus subtilis*, *Bifidobacterium bifidum*	*Acipenser baerii* AE (Fingerlings)	1 × 10^6^, 2 × 10^6^, 3 × 10^6^ CFU/g for 56 days	10.50 ± 0.14	Increase of lysozyme and IgM levels (optimum at 3 × 10^6^ CFU/g)	[199]
*Lactococcus garvieae*	*Oreochromis niloticus * FW	1 × 10^7^ CFU/g for 2 weeks	50.00 ± 5.00	Increase of IgM and lysozyme activity	[200]
*Lactococcus lactis*	*Paralichthys olivaceus * SW	1 × 10^9^ CFU/g for 8 weeks	80.84 ± 9.37	Enhancement of innate immune response and resistance to *Streptococcus*	[201]
*Lactococcus lactis*	*Cyprinus carpio* FW	5 × 10^8^ CFU/g for 8 weeks	33.07 ± 0.55	Increase of pro-inflammatory cytokines	[202]
*Lactococcus lactis*	*Sparus aurata * SW	2 × 10^9^, 5 × 10^9^ CFU/kg for 12 weeks	70.00–90.00	Increase of gene expression of immune responses	[9]
*Lactobacillus rhamnosus GG*	*Oreochromis niloticus * FW	1 × 10^8^ CFU/g for 2 weeks	14.05 ± 0.42	Increase of lysozyme activity, mucous cells at intestine and resistance to *Aeromonas veroni*	[203]
*Lactobacillus sakei PO11*, *Lb. plantarum PO23*	*Paralichthys olivaceus * SW	1 × 10^11^ CFU/g for 27 days	35.00 ± 5.00	Increase of gene expression of immune responses (interleukins and immunoglobulins)	[204]
*Bacillus subtilis* and *Saccharomyces cerevisiae*	*Oreochromis niloticus* FW	6 × 10^7^ CFU/g for 56 days	24.01 ± 0.02	Enhancement of innate immune responses and resistance to pathologies	[205]
*Paenibacillus ehimensis*	*Oreochromis niloticus* FW	1 × 10^6^, 1 × 10^7^ CFU/g for 60 days	5.53 ± 0.45	Enhancement of immune response and resistance to pathologies	[206]
*Paenibacillus polymyxa*	*Cyprinus carpio * FW	1 × 10^3^, 1 × 10^4^, 1 × 10^5^ CFU/mL for 8 weeks	23.17	Increase of lysozyme activity, myeloperoxidase, and resistance to *A. hydrophila * (optimum at 1 × 10^3^ CFU/mL)	[207]
*Pediococcus acidilactici*	*Oreochromis niloticus* FW	1 × 10^10^ CFU/kg for 32 days	175.00	Increase of lysozyme activity and leukocytes	[208]
*Pediococcus acidilactici*	*Oreochromis niloticus * FW	2.81 × 10^6^ CFU/g for 6 weeks	9.19 ± 0.04	Increase of leukocytes and intestinal mucous cells	[209]
*Pediococcus acidilactici*	*Cyprinus carpio * FW	6 × 10^8^ CFU/g for 60 days	10.00 ± 2.50	Increase of Ig and proteases of epithelial mucosa, gene expression of lysozyme and TNF-α	[210]
*Pediococcus pentosaceus*	*Ctenopharyngodon idella* FW	1 × 10^9^ CFU/g for 30 days	32.10 ± 9.00	Increase of gene expression of immune response and resistance to *A. hydrophila*	[211]
*Pseudomonas fluorescens biovar I*, *II* and *III*	*Oreochromis niloticus* FW	1 × 10^11^ CFU/kg for 45 days	2.93 ± 0.22	Enhancement of non-specific immune and blood parameters	[212]
*Psychrobacter namhaensis SO89*	*Oreochromis niloticus * FW	2.8 × 10^7^, 5.6 × 10^7^ for 50 days	4.58 ± 0.14	Increase of haematocrit, leukocytes and other immune parameters	[117]
*Saccharomyces cerevisiae*	*Pangasianodon hypophthalmus* FW	1 × 10^9^, 1 × 10^11^ CFU/kg for 120 days	55.00–65.00	Enhancement of immune parameters (immunglobulines, lysozyme activity)	[213]
*Saccharomyces cerevisiae*	*Oreochromis niloticus* FW	0.25, 0.50, 1, 2, 5 g/kg for 12 weeks	0.33	Enhancement of immune parameters and resistance to pathologies (optimum at 1 g/kg)	[214]
*Shewanella putrefaciens Pdp11*	*Sparus orata * SW	1 × 10^8^ CFU/g for 15–30 days	104.20 ± 7.40	Increase of lysozyme activity and gene expression of immune responses	[215]
*Shewanella xiamenensis*, *Aeromonas veronii*	*Ctenopharyngodon idella* FW	1 × 10^8^ cell/g for 28 days	35.00 ± 5.00	Enhancement of immune parameters, and resistance to *A. hydrophila*	[216]
*Vibrio lentus*	*Dicentrarchus labrax * SW (Larvae)	1 × 10^6^ CFU/mL for 10 days post-hatching		Increase of transcription of genes of immune responses	[217]
*Weissella confusa*	*Oncorhynchus mykiss* FW	1.5 × 10^10^, 3 × 10^10^, 4.5 × 10^10^ CFU/kg for 60 days	115.00 ± 2.60	Increase of lysozyme activity and expression of INF-γ, TNF-α and IL-8 at 3 × 10^10^ CFU/kg	[218]

**Table 6:** Administration of prebiotics enhancing the gut efficiency and growth performance in different aquaculture fish species. (SW—seawater; FW—freshwater; AE—anadromous euryhaline; MOS—mannan-oligosaccharides; GOS—galacto-oligosaccharides; COS—chito-oligosaccarides; FOS—fructo-oligosaccharides).

Prebiotics	Fish Species	Dosage and Timing of Administration	Initial Weight (g)	Effects	References
β-glucan + MOS	*Oreochromis niloticus* FW (Fingerlings)	1.5, 3 g/kg for 60 days	8.70 ± 0.40	Enhancement of growth performance and histological parameters of the intestine	[224]
β-glucan, GOS, MOS	*Channa striata * FW	2 g/kg (β-glucan), 5 g/kg (GOS), 5 g/kg (MOS) for 16 weeks	22.40	Enhancement of growth performance and protein digestibility	[225]
β-glucan + MOS	*Tor grypus * FW	1.5% (MOS), 1.5% (β-glucan) for 90 days	35.00 ± 1.20	Enhancement of growth performance and increase of protein content in fish	[226]
COS	*Scophthalmus maximus * SW	2 g/kg for 56 days	11.00	Enhancement of growth performance and positive effects on intestine structure	[129]
FOS	*Atractosteus tropicus * SW (Larvae)	7.5 g/kg for 15 days	0.03	Enhancement of growth performance and digestive capacity	[130]
FOS	*Sciaenops ocellatus * FW	1% for 8 weeks	7.00	Increase in height of intestinal microvilli	[152]
FOS	*Oreochromis niloticus * FW	0.5, 1, 2, 4 g/kg for 8 weeks	5.00 ± 0.02	Increase of amylase enzyme and height of anterior intestinal microvilli with enhancement of growth parameters (optimum at 1 g/kg)	[132]
GOS	*Sciaenops ocellatus * FW	1% for 8 weeks	7.00	Increase in height of intestinal microvilli	[152]
GOS	*Pangasius hypophthalmus* FW	10 g/kg for 12 weeks	16.45 ± 0.07	Increase of digestive enzymes activity and length of posterior intestinal microvilli	[227]
Inosine, monophosphate inosine	*Pagrus major * SW	0.2%, 0.4%, 0.6%, 0.8% for 10 weeks	6.60	Increase in height of enterocytes, intestinal folds and microvilli (optimum at 0.4%)	[228]
Inulin	*Sciaenops ocellatus * FW	1% for 8 weeks	7.00	Increase in height of intestinal microvilli	[152]
Inulin	*Lates calcarifer* FW	15, 20 g/kg for 60 days	7.14 ± 0.05	Increase of intestinal adsorbent surface	[141]
MOS	*Sciaenops ocellatus * FW	1% for 8 weeks	7.00	Increase in height of intestinal microvilli	[152]
MOS	*Ctenopharyngodon idella * FW	200, 400, 600, 800 and 1000 mg/kg for 60 days	215.85 ± 0.30	Enhancement of the intestinal health status in fish with enteritis by *Aeromonas hydrophila* (optimum at 400 mg/kg)	[229]
Nucleotides	*Sciaenops ocellatus * FW	1% for 6 weeks	7.10	Increase in height of intestinal folds and microvilli	[230]
Nucleotides (NucleoforceFish™)	*Sparus aurata * SW	250, 500 mg/kg for 150 days	0.36 ± 0.002	Enhancement of growth parameters	[154]
Essential oils of *Ocimum basilicum* (EOOB)	*Oreochromis niloticus * FW	0.25, 0.5, 1, 2 mL of EOOB/kg for 45 days	12.13 ± 0.11	Enhancement of digestive enzymes activity (amylases) (optimum at 1 mL/kg)	[219]
Stachyose	*Scophthalmus maximus* L. SW	1.25%, 5% for 12 weeks	4.63 ± 0.01	Increase of gene expression of occludins and ZO-1, and intestinal health (optimum at 5%)	[156]
Thymol and carvacrol	*Sparus aurata * SW	From 0.005 to 0.03% for 9 weeks	26.00–27.00	Increase in number of enterocytes e mucous cells in intestinal epithelium, and enhancement of feed gain ratio at 0.01%	[157]

**Table 7:** Administration of probiotics enhancing the gut efficiency and growth performance in different aquaculture fish species. (SW—seawater; FW—freshwater; AE—anadromous euryhaline).

Probiotics	Fish Species	Dosage and Timing of Administration	Initial Weight (g)	Effects	References
*Acinetobacter KU011TH*	*Clarias macrocephalus * FW	1 × 10^5^, 1 × 10^7^, 1 × 10^9^ CFU/kg and 1 × 10^3^, 1 × 10^4^, 1 × 10^5^ CFU/mL	150.00	Enhancement of growth performance at 1 × 10^9^ CFU/kg and 1 × 10^3^ CFU/mL	[231]
*Acinetobacter*, *Vibrio*, *Bacillus*, *Alcaligens*, *Photobacterium*, *Flavobacterium*	*Oncorhynchus mykiss * FW	3.9 × 10^9^ CFU/g for 56 days	113.00 ± 10.40	Enhancement of growth performance	[232]
*Bacillus amyloliquefaciens*	*Oreochromis niloticus* FW	60 mg/kg for 42 days	39.00	Enhancement of growth performance	[233]
*Bacillus coagulans*, *Rhodopseudomonas palustris*, *Lactobacillus acidophilus*	*Ctenopharyngodon idella* FW	1 × 10^6^ CFU/g for 60 days	2.10 ± 0.09	Increase in weight and digestive enzymes activity	[234]
*Bacillus licheniformis*	*Cyprinus carpio * FW	1 × 10^6^, 1 × 10^7^, 1 × 10^8^ CFU/g for 60 days	38.58 ± 0.42	Enhancement of growth performance and increase in height of intestinal microvilli (optimum at 1 × 10^8^ CFU/g)	[163]
*Bacillus thuringiensis*, *Bacillus cereus*	*Lates calcarifer* FW	1 × 10^12^ CFU/kg for 42 days	50.00 ± 0.50	Enhancement of growth performance	[174]
*Bacillus velezensis* *TPS3N*, *Bacillus subtilis TPS4*, *Bacillus amyloliquefaciens*	*Oreochromis niloticus * FW	1 × 10^8^ CFU/mL for 4 weeks	46.24 ± 0.48	Enhancement of digestive enzymes activity and increase in thickness and length of intestinal microvilli, and number of mucous cells	[175]
*Bacillus* sp.	*Cyprinus carpio* FW	~1 × 10^11^ CFU/g for 60 days	5.90–7.10	Enhancement of digestive enzymes activity and food conversion rate	[235]
*Bacillus* sp., *Alcaligenes* sp.	*Tor tambroides* FW	1 × 10^8^ CFU/g for 90 days	1.39 ± 0.06	Enhancement of growth rate and digestive enzymes activity, and positive effects on intestinal morphology	[236]
*Bacillus* sp*. SJ-10*, *Lactobacillus plantarum*	*Paralichthys olivaceus * SW	1 × 10^8^ CFU/g for 8 weeks	14.92 ± 0.21	Not significant enhancement of some histo-morphological intestinal parameters and increase in some digestive enzymes activity	[237]
*Bacillus subtilis*	*Oreochromis niloticus* FW	1.1 × 10^5^ CFU/g for 84 days	5.26 ± 0.06	Enhancement of growth performance	[181]
*Bacillus subtilis* and *Lactobacillus rhamnosus*	*Labeo rohita * FW (Fingerlings)	1 × 10^7^ CFU/g for 60 days	0.38 ± 0.015	Enhancement of growth performance and nutrients usage	[238]
*Bacillus subtilis*, *Bacillus licheniformis*, *Bacillus pumilus*	*Oreochromis niloticus* FW	20 mg/kg for 49 days	34.56 ± 0.05	Increase in number of microvilli and lysozyme activity, no changes in growth performance and blood and immune parameters	[239]
*Bacillus subtilis*, *Bacillus licheniformis*, *Bacillus pumilus*	*Panganodon hypophthalmus* FW	1 × 10^10^ CFU/g for 110 days	0.03	Enhancement of growth performance and survival rate	[240]
*Bacillus*, *Bifidobacterium*, *Enterococcus*, *Lactobacillus*, *Pediococcus* sp.	*Oreochromis niloticus * FW (Fingerlings)	1, 2 g/kg for 90 days	5.00	Increase in final weight and muscle mass	[241]
*Enterococcus casseliflavus* *(EC-001)*	*Cyprinus carpio* FW	1 × 10^10^, 1 × 10^11^, 1 × 10^12^ CFU/kg for 56 days	12.00 ± 0.50	Increase in weight at 1 × 10^11^ and 1 × 10^12^ CFU/kg	[184]
*Enterococcus faecium*	*Oreochromis niloticus * FW	1 × 10^7^ CFU/mL for 40 days	6.83 ± 0.18	Increase in final weight	[116]
*Lactobacillus. acidophilus*	*Cyprinus carpio* FW	1 × 10^2^, 1 × 10^4^, 1 × 10^6^ CFU/kg for 56 days	21.34 ± 1.85	Enhancement of length and width of microvilli of growth performance (optimum at 1 × 10^6^ CFU/kg)	[192]
*Lactobacillus rhamnosus ATCC 7469*	*Oncorhynchus mykiss* FW	1 × 10^9^ CFU/kg for 60 days	18.41 ± 0.32	Enhancement of growth parameters	[189]
*Lactobacillus acidophilus*	*Xiphophorus helleri* FW	1.5 × 10^8^, 3 × 10^8^, 6 × 10^8^ CFU/g for 70 days	3.90 ± 0.20	Enhancement of growth performance (optimum at 6 × 10^8^ CFU/g)	[190]
*Lactobacillus casei*	*Cyprinus carpio * FW	1 × 10^8^ CFU/g for 30 days	68.40 ± 5.90	Enhancement of intestinal enzyme parameters	[242]
*Lactobacillus casei* (Yakult^®^)	*Tor tambra* FW (Larvae)	0, 5, 10, 15 mL/kg for 80 days	0.03	Enhancement of growth performance and nutrients usage (optimum at 10 mL/kg)	[243]
*Lactobacillus delbrueckii*	*Poecilia sphenops* FW	0.8 g/kg for 30 days	0.90 ± 0.02	Enhancement of growth performance and survival rate	[244]
*Lactobacillus plantarum*	*Oreochromis niloticus* FW	1 × 10^8^ CFU/g for 84 days	5.92 ± 0.08	Enhancement of growth performance	[196]
*Lactobacillus. rhamnosus GG*	*Oreochromis niloticus * FW	1 × 10^8^ CFU/g for 2 weeks	14.05 ± 0.42	Enhancement of histo-morphological intestinal parameters	[203]
*Lactobacillus rhamnosus JCM1136*, *Lactobacillus lactis* subsp. *lactis JCM1136*	*Oreochromis niloticus * FW	1 × 10^8^ CFU/g for 6 weeks	0.20 ± 0.05	Increase in length and density of intestinal microvilli	[198]
*Micrococcus luteus*	*Oreochromis niloticus* FW	1 × 10^7^ cells/g for 90 days	2.35 ± 0.10	Enhancement of growth performance	[245]
*Micrococcus MCCB 104*, *Bacillus MCCB 101*	*Oreochromis mossambicus* FW	1 × 10^3^ CFU per individual for 28 days		Increase of intestinal and hepato-pancreatic enzymes activity	[246]
*Pediococcus acidilactici*	*Oreochromis niloticus* FW	1 × 10^10^ CFU/kg for 32 days	175.00	No significant change in growth parameters	[208]
*Pediococcus pentosaceus SL001*	*Ctenopharyngodon idella * FW	1 × 10^9^ CFU/g for 30 days	32.10 ± 9.00	Increase in number of goblet cells and length of intestinal villi	[211]
*Pseudomonas fluorescens biovar I*, *II* and *III*	*Oreochromis niloticus* FW	1 × 10^11^ CFU/kg for 45 days	2.93 ± 0.22	Enhancement of growth performance and blood and non-specific immune parameters	[212]
*Saccharomyces cerevisiae*	*Pangasianodon hypophthalmus* FW	1 × 10^9^, 1 × 10^11^ CFU/kg for 120 days	55.00–65.00	Enhancement of growth rate, food conversion rate and immune parameters	[213]
*Saccharomyces cerevisiae* (Angel Yeast Co. Ltd., China)	*Oreochromis niloticus* FW (Fingerlings)	0, 1, 2, 4 g/kg for 60 days	7.55 ± 1.25	Increase of growth performance and change of histo-morphological intestinal parameters (optimum at 4 g/kg)	[247]
*Saccharomyces cerevisiae*	*Labeo rohita* FW (Fingerlings)	0, 1, 2, 4 g/kg for 90 days	5.69 ± 0.02	Enhancement of growth rate, histological parameters and nutrients usage (optimum at 4 g/Kg)	[248]
*Saccharomyces cerevisiae*	*Mystus cavasius* FW	0, 0.5, 1, 1.5 g/kg for 75 days	0.50 ± 0.20	Enhancement of growth performance (optimum at 1 g/kg)	[249]
*Streptomyces* spp.	*Xiphophorus helleri* FW	10 g/kg for 50 days	0.60	Enhancement of growth performance and nutrients usage	[250]

**Table 8:** The impact of some prebiotics/probiotics on the gut of aquaculture fish species assessed by histological endpoints. (↑: increase; ↓: decrease; =: no change).

Prebiotics/Probiotics	Fish Species	Histological Endpoints	References
Lactic acid bacteria Saccharomyces	*Oreochromis niloticus*	↑ intestinal perimeter ratio, mucosal fold length, goblet cells and leukocytes	[222]
*P. acidilactici MA18/5M* + short-chain fructo-oligosaccharides	*Salmo salar*	↑ mucosal fold height, intraepithelial leukocytes, granulocytes, and goblet cells	[258]
*Bacillus coagulans*, *B. licheniformis* and *Paenibacillus polymyxa*	*Sillago sihama*	↑ villus height, villus width, muscle thickness, and crypt depth	[223]
*Bacillus subtilis +* inulin	*Sparus aurata L.*	↑ enterocyte vacuolisation, microvilli disruption	[221]
*Lactococcus lactis*	*Sparus aurata*	= mucosal folds, connective tissue, lamina propria of simple folds, supranuclear vacuoles	[9]
Nucleotides	*Sciaenops ocellatus*	↑ microvillus height, enterocyte height, = fold height	[230]
*Lactobacillus brevis* and *L. buchneri*	*Seriola dumerili*	↓ thickness of submucosa layer	[259]
*Bacillus licheniformis*, *Bacillus subtilis + Saccharomyces cerevisiae*	*Acipenser persicus*	↑ goblet and mucosal cell number ↑ villus length and width	[260]
*Aquablend^®^ + GroBiotic^®^*	*Totoaba macdonaldi*	↑ fold length, = fold width, = enterocyte height, = microvilli height	[261]
Short-chain fructo-oligosaccharides and xylo-oligosaccharides	*Dicentrarchus labrax*	↓ mucosal folds, = enterocytes nucleous, ↑ supranuclear absorptive vacuolisation, = hyperplasia in the lamina propria and submucosa, ↑ intraepithelial leucocytes	[262]
*Bacillus velezensis TPS3N*, *Bacillus subtilisTPS4*, *Bacillus amyloliquefaciens TPS17* (as singular and combined suspension)	*Oreochromis niloticus*	↑ villus length and width, goblet cells count, intestinal epithelial muscle thickness	[175]
AquaStar^®^ Growout *(Lactobacillus reuteri*, *Bacillus subtilis*, *Enterococcus faecium* and *Pediococcus acidilactici)*	*Oreochromis niloticus*	↑ intraepithelial leucocytes, microvilli density	[172,209,263]

## Data Availability

The data presented in this study are available on request from the corresponding author.
